# Defect detection of gear parts in virtual manufacturing

**DOI:** 10.1186/s42492-023-00133-8

**Published:** 2023-03-29

**Authors:** Zhenxing Xu, Aizeng Wang, Fei Hou, Gang Zhao

**Affiliations:** 1 School of Mechanical Engineering & Automation, Beihang University, Beijing, 100191 China; 2 State Key Laboratory of Computer Science, Institute of Software, Chinese Academy of Sciences, Beijing, 100190 China; 3 University of Chinese Academy of Sciences, Beijing, 100049 China

**Keywords:** Defect detection, Gear surface, Gear dataset, Combinational Convolution Block

## Abstract

Gears play an important role in virtual manufacturing systems for digital twins; however, the image of gear tooth defects is difficult to acquire owing to its non-convex shape. In this study, a deep learning network is proposed to detect gear defects based on their point cloud representation. This approach mainly consists of three steps: (1) Various types of gear defects are classified into four cases (fracture, pitting, glue, and wear); A 3D gear dataset was constructed with 10000 instances following the aforementioned classification. (2) Gear-PCNet+ + introduces a novel Combinational Convolution Block, proposed based on the gear dataset for gear defect detection to effectively extract the local gear information and identify its complex topology; (3) Compared with other methods, experiments show that this method can achieve better recognition results for gear defects with higher efficiency and practicability.

## Introduction

Virtual manufacturing is a simulation-based technology for defining, simulating, and visualizing the manufacturing process in the design stage. During manufacturing, product defect detection is closely related to quality assurance. The detection of 3D objects has been widely studied [1–4]. Mechanical gears are widely used in the power transmission of various industrial machinery, including turbines, motor vehicles, and aircraft [5]. Gear defect detection is crucial in virtual manufacturing to detect faults incurred during the manufacturing simulation. However, gear defects are inevitable in an actual industrial environment with almost 80% of the faults in mechanical transmission systems caused by gear defects [6], resulting in manufacturing and financial losses, in addition to personal safety issues. Thus, defect detection is necessary in mechanical systems.

Traditionally, researchers artificially collected the characteristics of vibration and acoustic emission signals to monitor the condition of rotating machinery [7]. Signal-based methods [1–3] are also effective for gears, but they often require accurate physical models and signal processing experience [8, 9], which are insufficient to satisfy the modern industry requirements of intelligence. Sensor data were the basis for detection. Li et al. [10] collected information from different sensors to analyze defect features. However, the defect vibration signals were acquired by running the gear and the defects may be submerged in strong meshing harmonics of various rotary components.

Deep learning has great advantages in image classification [11–13] and target detection [14, 15] owing to its feature extraction and nonlinear approximation abilities. Furthermore, intelligent data-driven fault diagnosis technology has been receiving more attention. Li et al. [16] proposed a separation-fusion-based deep learning approach to analyze multi-modal features of gearbox vibration measurements and obtained the results of diagnosis. For traditional methods, modulated signals of gears are impractical in extracting features and detecting defects. On the other hand, image-based computer vision can be used in defect detection [17]. Researchers tried to use 2D images of gears to recognize gear defects [18] besides the gray image transformed by vibration signals [19, 20]. Nonetheless, it is difficult to recognize the defects of gears, especially on the tooth surface owing to its complex concave structure. In addition, the textures of oil stains or rust on gear surfaces with the results from images and cause confusion in defect detection [21].

Compared with the image-based methods, 3D point cloud models with depth data can avoid the misrecognition of gear defects from image texture or oil marks. Charles et al. [22] first proposed a network of point clouds: PointNet. Then, various point cloud-based deep learning networks were successfully used in 3D shape classification, object detection, tracking, and 3D segmentation [23, 24]. Massive, labeled data with defect information is the key to ensuring good detection performance of neural networks. Nevertheless, it is difficult to collect adequate data for the machines, which is a limiting factor for intelligent fault diagnosis. Researchers tackled the issue of lack of labeled data by transfer learning [25, 26] and semi-supervised/unsupervised learning [27] methods. However, a noise-free point cloud can be obtained from the computer aided design (CAD) model of a gear through virtual manufacturing. This makes it significant in checking the defect detection results using point cloud data. Besides, gear model with defects has complex local structures that can be fully represented by point clouds. Therefore, in this study, a new artificial neural network, Gear-PCNet++, is presented based on point clouds extracted from CAD models. In this network, a novel Combinational Convolution Block (CCB) is proposed to replace the convolution layer in Multi-Layer Perception (MLP) networks to extract more gear defect details.

The main contributions of this study are: (1) construction of a data set of 3D gear models, which has 4 typical gear defects: fracture, pitting, glue, and wear; (2) CCB combining multi-level features of gears, which improves the precision rate of defect detection; (3) development of a new network, Gear-PCNet++, based on CCB, enabling gear defects of various types to be recognized with high accuracy.

## Methods

### Construction of 3D gear sample sets

The data of point clouds can be obtained from 3D scanning, but it is difficult to accurately label the categories for scanned raw point clouds. Based on the geometric properties of gears, an approach for 3D gear data generation is proposed.

**Fig. 1:**
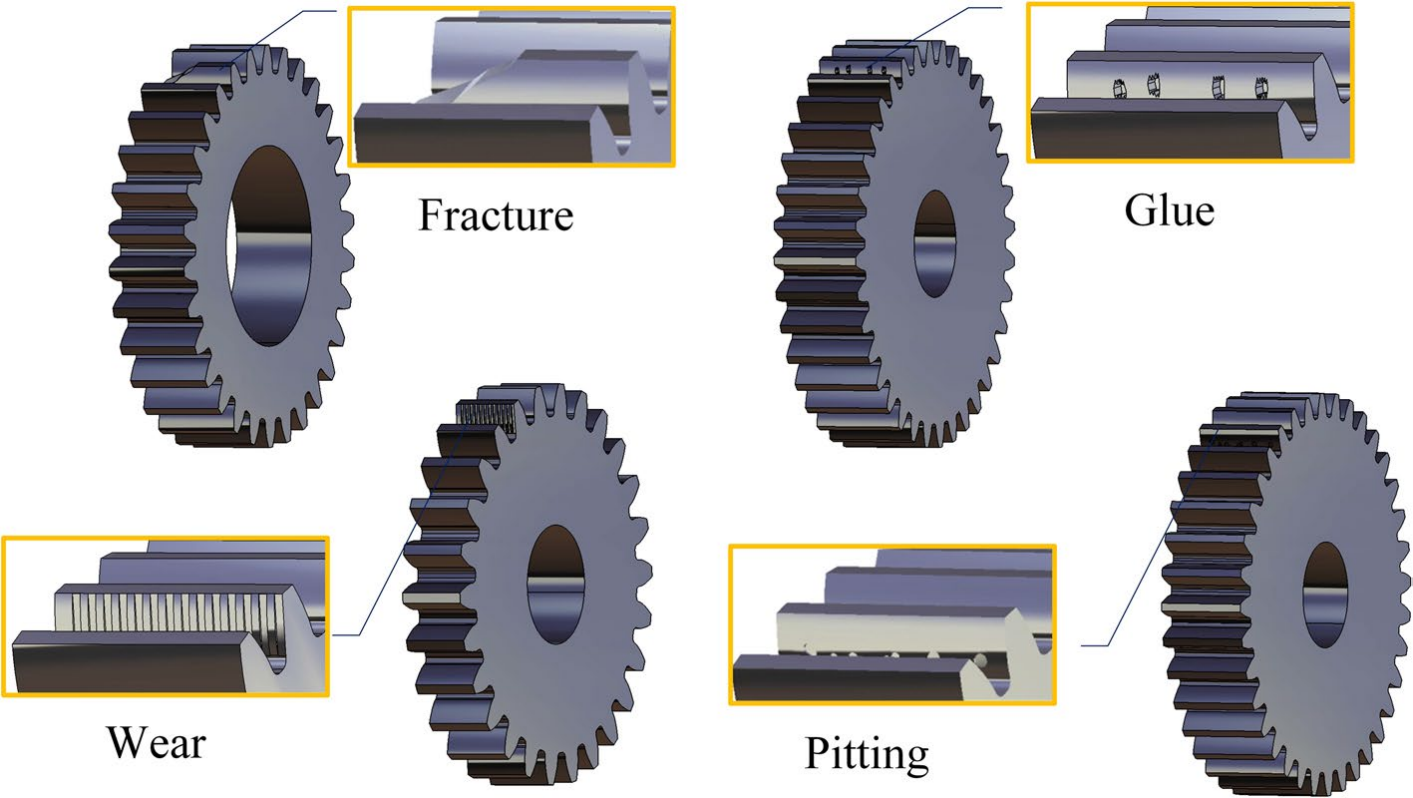
Four typical gear defects

In this study, gear defects are classified into four typical types: wear, pitting, glue, and fracture [5]. The gear defects can be represented as a combination of the four typical defects, as illustrated in Fig. 1.

Five basic gears were constructed with different parameters: modulus, tooth number, tooth width, and diameter of center hole (Table 1).

**Table 1:** Parameters of basic gears

Substrate symbol	Tooth number	Modulus (mm)	Tooth width (mm)	The diameter of center hole (mm)
1	18	4.00	15.00	20.00
2	22	2.00	8.00	15.00
3	26	3.00	13.00	25.00
4	30	2.50	16.00	40.00
5	36	2.00	17.00	20.00

Let W, P, G, and B represent wear, pitting, glue, and fracture, respectively, and S denote normal gear (basic gear). The gear models with defects are generated by combining defects and the basic gear using Eq. 1.

1 $$Gr_{def,i} = Gr_{bas,j} + Def_{i} = \{ Base,W,P,B\} ,$$

Where *Gr*_*def,i*_ is the *i*-th generated gear with defects, *Gr*_*bas,j*_ is the *j*-th basic gear, and *Def*_*i*_ is the defects of the *i*-th generated gear.

**Fig. 2:**
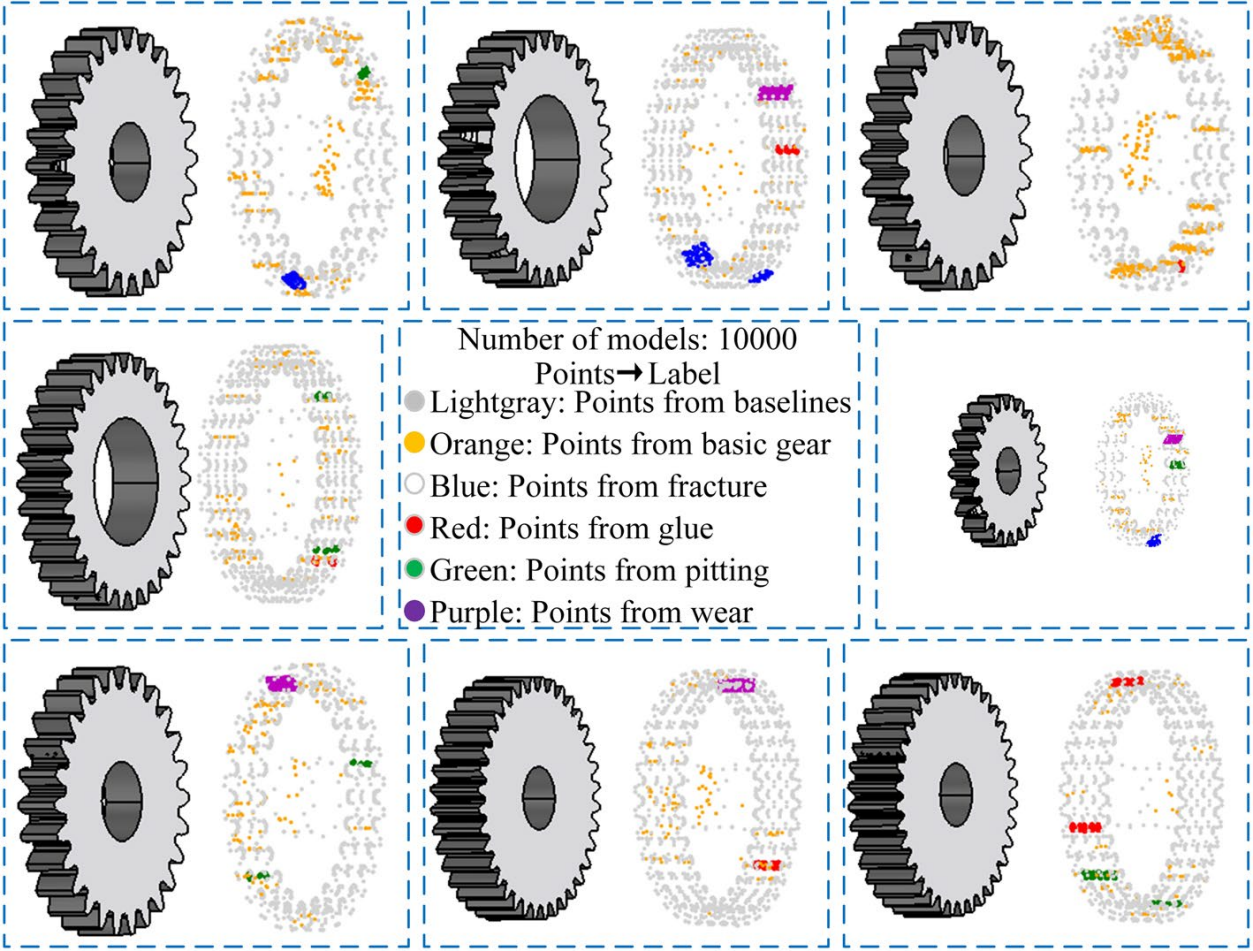
Gear data set with defects. The light gray, orange, blue, red, green, and purple spheres represent points of baselines, basic gear, fracture, glue, pitting, and wear, respectively

The CAD model is transferred into a point cloud model. Though the CAD model of the gear has a large number of surface elements, effective surfaces are randomly used to discretize points. Finally, a point cloud data set with 10000 gear samples is constructed; some of which are shown in Fig. 2.

### CCB

**Fig. 3:**
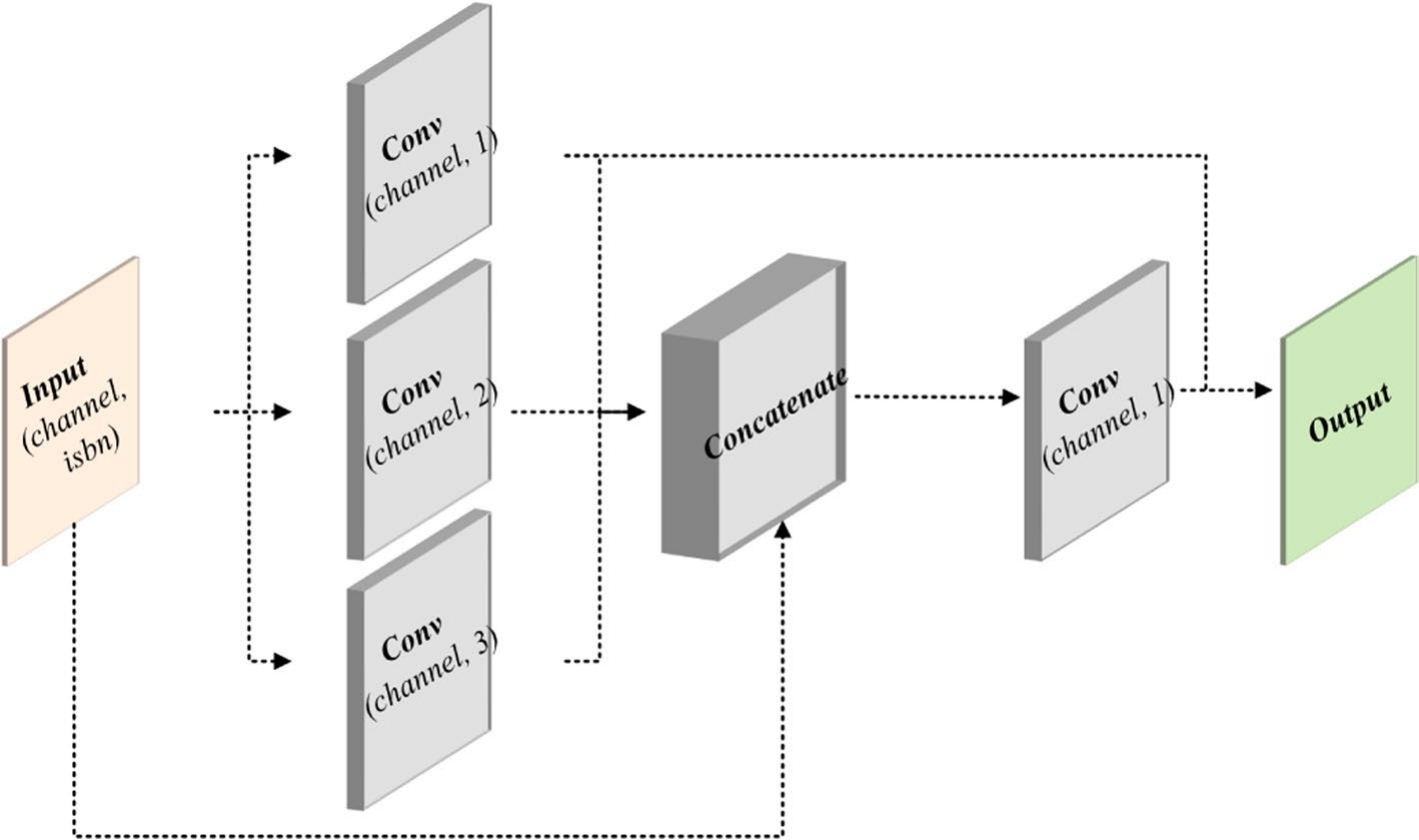
CCB. Channel represents the number of channels of input feature vector. Isbn represents whether to add batch normalization to each convolution layer

The location of gear defects occurred on the tooth surface is frequently similar. Hence, it is difficult to identify gear defects from the local features of point clouds. The boundary information is more critical than other details for gears [22, 28]. The CCB module is proposed (Fig. 3) to improve the ability to identify gear features, especially the boundary lines.

A convolution layer significantly improves the efficiency of parameters by sharing weights and is widely used in artificial neural networks. Wu et al. [29] proposed PointConv using Monte Carlo approximation. This architecture is a convolution operation suitable for unstructured point cloud data. It has also been verified that dilated convolution and down sampling were effective ways to expand receptive fields [30]. Dilated Point Convolutions uses K·D nearest neighbors to replace the original k-nearest neighbor partition [31], and extracts the features of each *d*-th point. With the same parameter, it increases the receptive field of PointConv. This is similar to dilated convolution, but it may lead to loss of details with local features. PointNet++ uses neighborhood-based feature extraction to replace the independent learning of each point [32], notably overcoming the limitations of PointNet [22]. Inspired by Deformable CNN [33], the Deformable KPconv in ref. [34] assigns different convolution kernels to each local geometry.

The receptive fields play an important role in semantic segmentation. Essentially, the size of receptive fields is related to the number of convolution layers and the size of convolution kernels. For deeper networks, larger kernel size corresponds to larger receptive fields but large convolution kernels may cause performance degradation. The sizes of convolution kernels typically used in structured data images are $3 \times 3$, $5 \times 5$, or $7 \times 7$. For unstructured point cloud models, a large convolution kernel will extract a lot of useless inter-point or point-domain information, which may be trivial to the improvement of performance. Multi-scale analysis is another strategy to improve the effect in image semantic segmentation [35–37], which can also enrich feature information. In addition, feature pyramid networks [38] is the most commonly used framework. Based on the above multi-scale or multi-level information interaction, this multi-scale synthesis strategy is applied to the convolution and uses a relatively small convolution kernel to obtain feature-rich information. Specifically, convolution kernels with different sizes are used to extract features under different receptive fields, and are then connected to the result of this module. The convolution of $1 \times 1$ has been widely used in ResNet, GoogLeNet [39], and other architectures. In the aforementioned module, $1 \times 1$ convolution is also used to achieve dimensional transformation to reduce the number of parameters. Moreover, the selection of convolution kernel size is based on the ideas discussed further.

Points, lines, and faces are the basic geometrical elements of gears. Two and three points can determine the corresponding line and plane, respectively; the point cloud is sparse relative to the original 3D model. It is assumed that a surface contains at least three points, of which two form a boundary line in a point cloud of gears. Then, the relevant geometric element information is extracted using kernel sizes 1, 2, and 3, and the corresponding features can be identified as projection points, pseudo lines, and pseudo surfaces, respectively, to a certain extent.

**Fig. 4:**
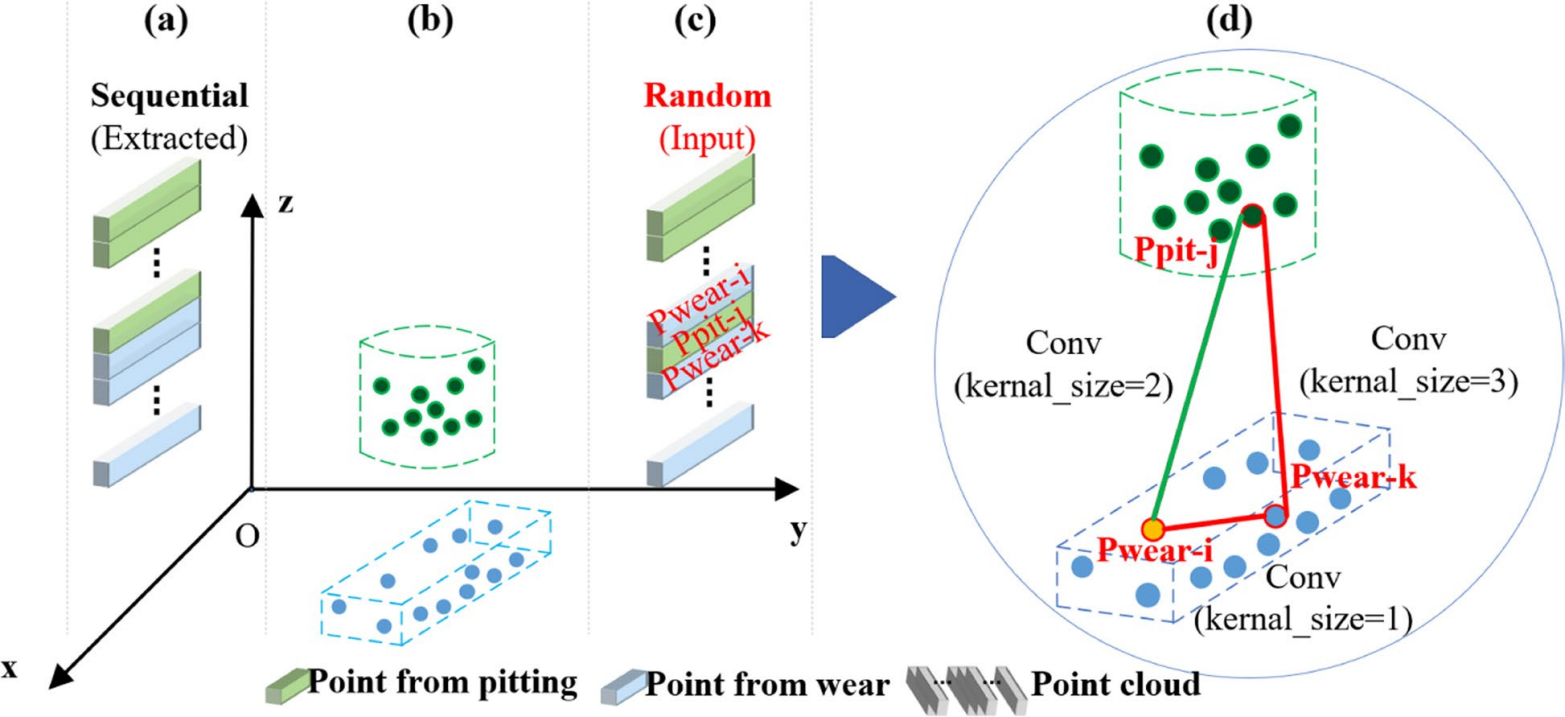
Ordered/unorganized point cloud and the receptive field corresponding to different convolution kernel sizes. **a** The ordered point cloud extracted from the gear discretization; **b** Result of dispersing pitting defects and wear defects into point clouds; **c** The point cloud, result of dispersing pitting defects and wear defects, input into the neural network after random shuffle; **d** Representation of the receptive field corresponding to different convolution kernel sizes in the network. In the circular region **d**, the orange dot represents the convolution of kernel size 1, the green line represents the convolution of kernel size 2, and the red triangle represents the convolution of kernel size 3

The point clouds in the input network are usually disordered. As shown in Fig. 4, there are pitting and wear defects in a gear, which are represented by green and blue cuboids, respectively. *P*_*pit-j*_, *P*_*wear-i*_, and *P*_*wear-k*_ are the points in pitting and wear, respectively. As for the point *P*_*wear-i*_, the large convolution kernel can easily extract the feature that makes little contribution to the point.

**Fig. 5:**
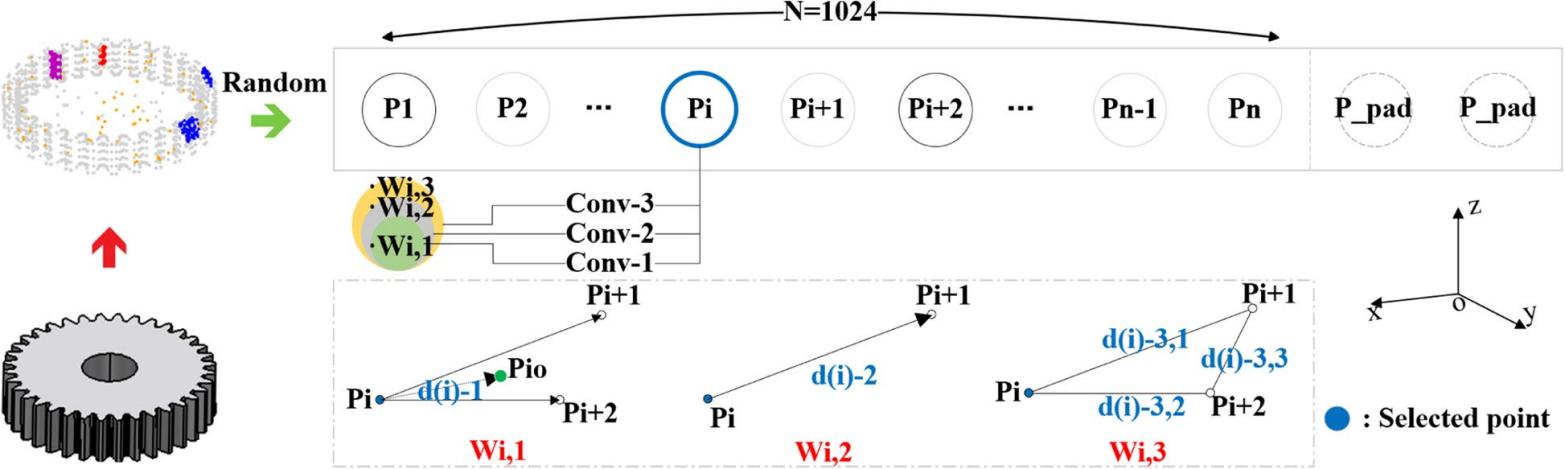
Feature weights optimization based on distance. $Conv_{i}$ is the convolution with kernel size *i*. $W_{i,j}$ is the weight of the *i*-th point and $j$ represents the size of kernel

To ensure the effectiveness of the extracted feature, defining the neighborhood of a point based on distance is a general strategy, which has been applied in many networks such as PointNet++, SpiderCNN [40], and EdgeConv [41]. Because of the difference between the study herein and the above methods a distance-based optimization strategy (Fig. 5) is proposed to assign corresponding weights to the features extracted by convolution kernels of different sizes.

The input point cloud is set as ${\mathrm{\{ }}p_{0} {,}p_{1} {,} \cdots p_{n} {\mathrm{\} }}$. Taking $p_{i}$ as an example, the extracted feature is related to three points: $p_{i}$,$p_{i + 1}$,$p_{i + 2}$ whose three-dimensional geometric center is $p_{i0} = \left( {p_{i} + p_{i + 1} + p_{i + 2} } \right)/3.$.

$W_{i,j}$ represents the weight of the *i*-th point whose convolution kernel size is $j$. When the convolution kernel is 2, the distance between the two points is directly related to the kernel. Thus, $W_{i,2\;}=k_2\;e^{-\vert p_ip_{i+1}\vert_d}$ can represent the corresponding weight. Similarly, when the convolution kernel is 3, the weight can be evaluated by a girth-related function:$W_{i,3}=k_3\;e^{-\left(\vert p_ip_{i+1}\vert_d+\vert p_ip_{i+2}\vert_d+\vert p_ip_{i+2}{\vert_d\vert}\right)/3}$.

From the above definition, it is obvious that the proportion of inter point features will decrease with the discretization of points. Therefore, a compensation coefficient $k$ is added to each weight to extract more local information. Furthermore, an eccentricity coefficient ($W_{i,1} = k_{1} \cdot e^{{ - \left| {p_{i} p_{io} } \right|_{d} }}$) is added when the convolution kernel is 1. The distance between *p*_*i*_ and *p*_*io*_ will decrease the proportion of the projection features of the point.

Through this multi-scale information synthesis, the proposed module can extract richer local features, and the latest extracted feature can be expressed using Eq. 2.

2 $$F_{i,com\_block} = F_{i,1} + Conv_{i} \left( {F_{i,ori} ,W_{i,1} \cdot F_{i,1} ,W_{i,2} \cdot F_{i,2} ,W_{i,3} \cdot F_{i,3} } \right)$$

Where *F*_*i,com_block*_ is the output feature of the module; *Conv*_*i*_ is the dimension transformation; *F*_*i,ori*_ is the input feature; *F*_*i,k*_ and *W*_*i,k*_ are the extracted feature and corresponding weight coefficient, respectively, when the convolution kernel is *k*. No additional weight calculation operation is required if the neighborhood is defined based on the distance.

### Network architecture

**Fig. 6:**
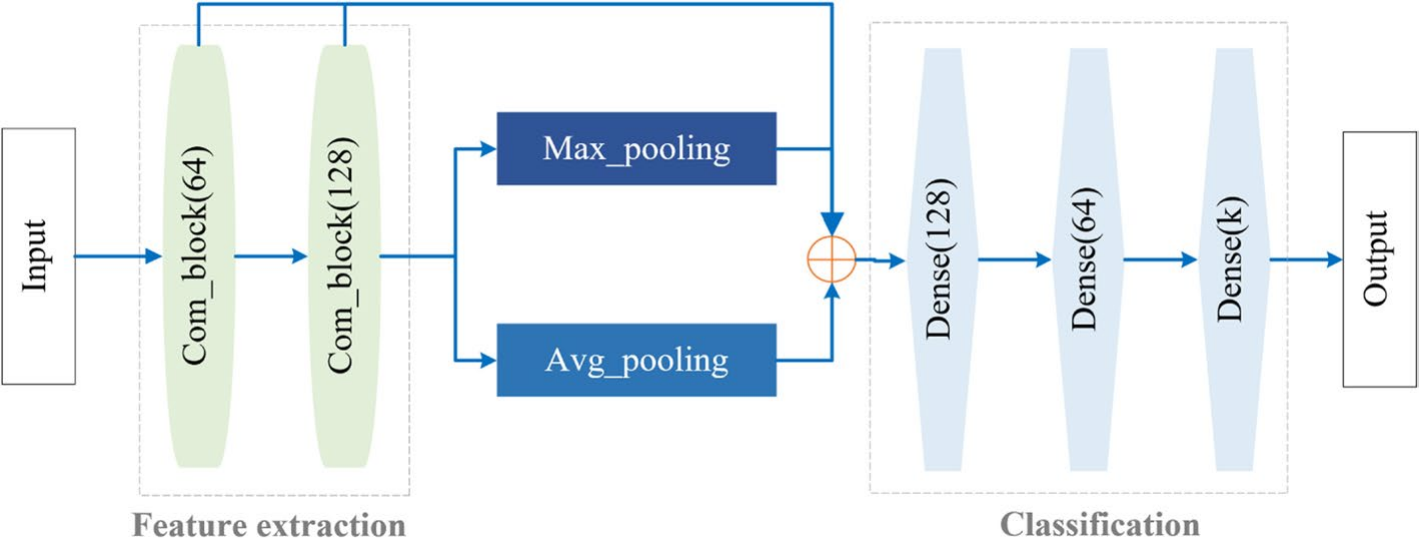
Structure of Gear-PCNet where the number is output channel of the layer

First, a gear defect recognition network based on 1D convolution operation is proposed: Gear-PCNet (Fig. 6). The network is composed of feature extraction (CCB-MLP) and final classification modules. Gear-PCNet can learn the representation of gear defects and output their results.

CCB can output features containing both single and inter-point information. The point cloud is the rotation and translation invariance. Projecting the point cloud data into 2D images or expressing it as voxels may lead to information loss. In PointNet, Charles et al. [22] dealt with the above two issues using the maximum value (Eq. 3). In Gear-PCNet, both the maximum and average functions are used (Eq. 4) to extract the features of point clouds and concatenate them.

3 $${\mathrm F}_{\max}=\mathrm{Max}\left({\mathrm x}_1,\;{\mathrm x}_2,\;...\;,\;{\mathrm x}_{\mathrm n}\right)$$

4 $${\mathrm F}_{\mathrm{avg}}=\frac{{\mathrm x}_1+{\mathrm x}_2+...+{\mathrm x}_{\mathrm n}}{\mathrm n}$$

After pooling and lateral linking the comprehensive features obtained by CCB-MLP, the classification of detected point cloud data can be completed through a fully connected network with three layers.

**Fig. 7:**
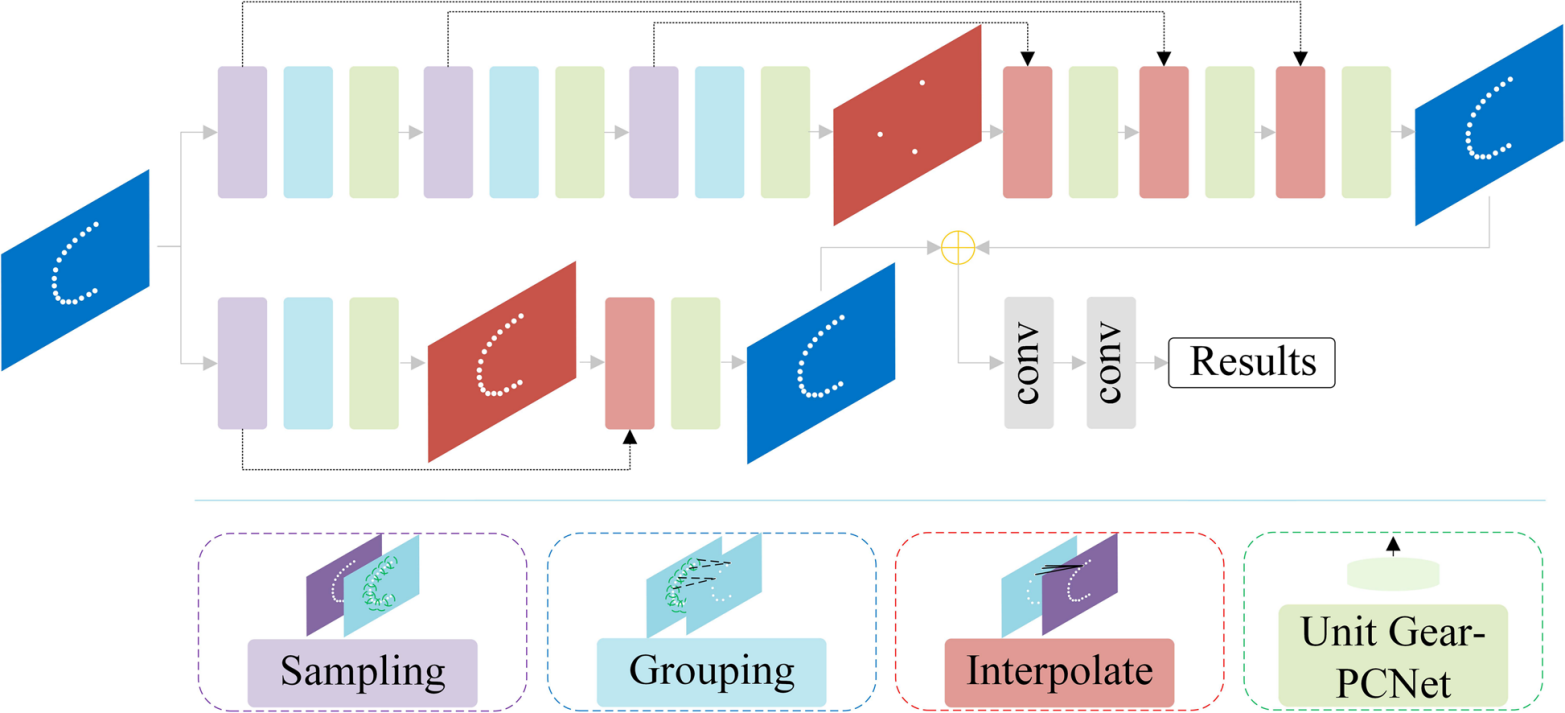
Structure of Gear-PCNet+ +

As verified above, the hierarchical feature learning framework is further applied to Gear-PCNet and Gear-PCNet++ is built based on the 2D convolution operation; the structure of Gear-PCNet++ is shown in Fig. 7. By constructing local region sets, the data set is relatively more concentrated, allowing the radius of local regions to be set small.

**Fig. 8:**
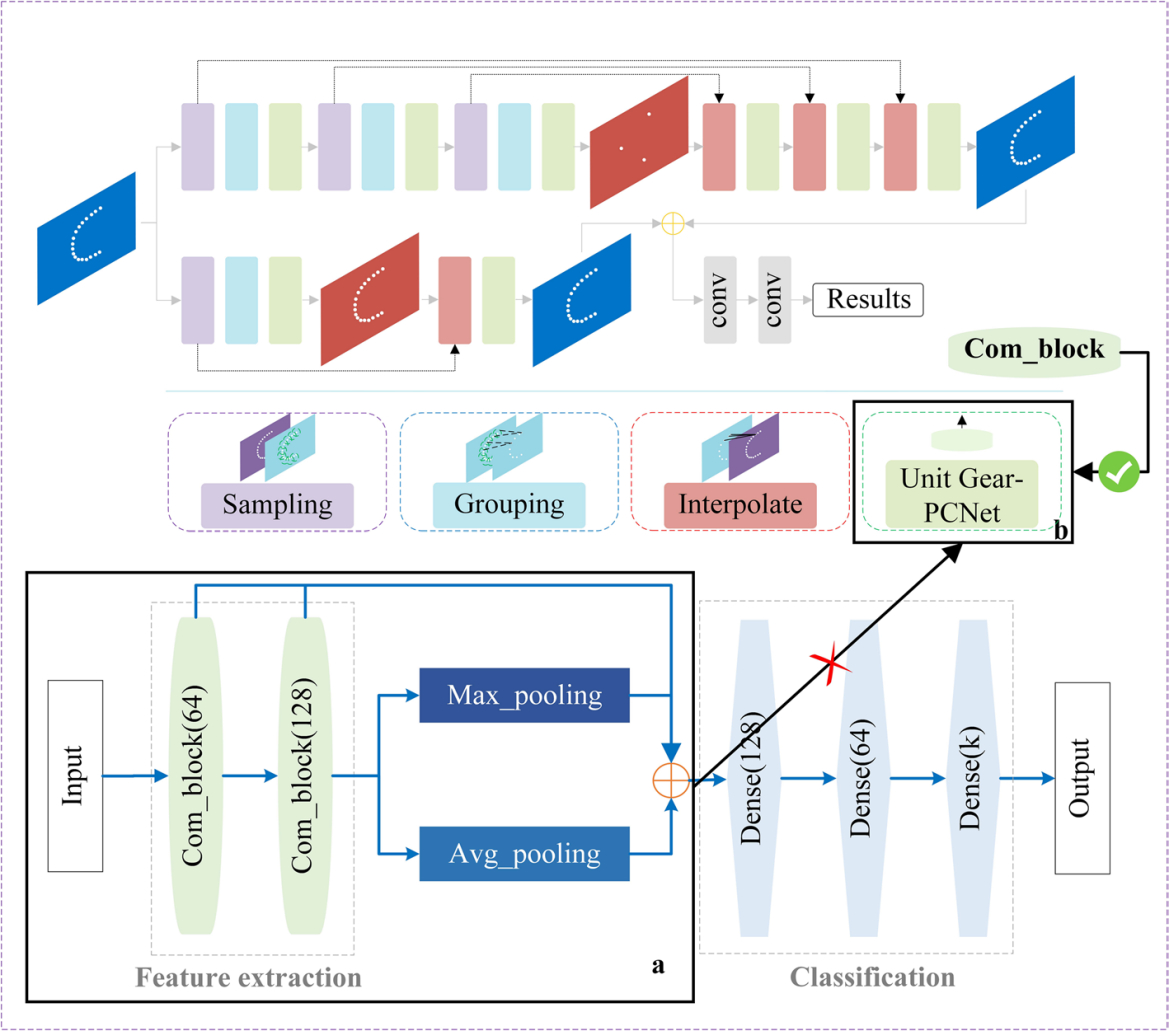
Feature extraction module in Gear-PCNet/Gear-PCNet+ +. **a** Feature extraction architecture in Gear-PCNet; **b** Feature extraction architecture in Gear-PCNet+ +

Unlike PointNet and PointNet++, Gear-PCNet was replaced with CCB to extract feature in Gear-PCNet++ (Fig. 8).

By using multi-resolution grouping, the two grouped features were propagated to the original points. Then, the two features were concatenated and regarded as the basis for point set segmentation.

## Results and Discussion

This approach was evaluated on a set of 10000 samples (gears with defects); their features can be grouped into 5 types: basic gear, fracture, pitting, glue, and wear. The 10000 samples were divided into training, validation, and testing sets in a 8:1:1 ratio, and experiments were run on a PC with a “NVIDIA GeForce RTX 3070” GPU and an “Intel Core i5-10400F @ 2.90GHz” CPU.

### Experiment results

The CCB is applied to Gear-PCNet to synthesize the features extracted under different convolution kernels. The number of parameters in Gear-PCNet is given in Table 2.

**Table 2:** Parameters in Gear-PCNet

Layer	Input channels	Kernel size	Stride	Output channels	Number of parameters
Com1_Conv1(+ BN1)	N × 3	1	1	N × 64	256 (+ 256)
Com1_Conv2(+ BN2)	N × 3	2	1	N × 64	448 (+ 256)
Com1_Conv3(+ BN3)	N × 3	3	1	N × 64	640 (+ 256)
Com1_Conv4(+ BN1)	N × 195	1	1	N × 64	12544 (+ 256)
Com2_Conv1(+ BN1)	N × 64	1	1	N × 128	8320 (+ 512)
Com2_Conv2(+ BN2)	N × 64	2	1	N × 128	16512 (+ 512)
Com2_Conv3(+ BN3)	N × 64	3	1	N × 128	24704 (+ 512)
Com2_Conv4(+ BN1)	N × 448	1	1	N × 128	57472 (+ 512)
Dense1	N × 579	-	-	N × 128	74240
Dense2	N × 128	-	-	N × 64	8256
Dense3	N × 64	-	-	N × 5	325
Total (trainable + non-trainable)					205253 + 1536

**Fig. 9:**
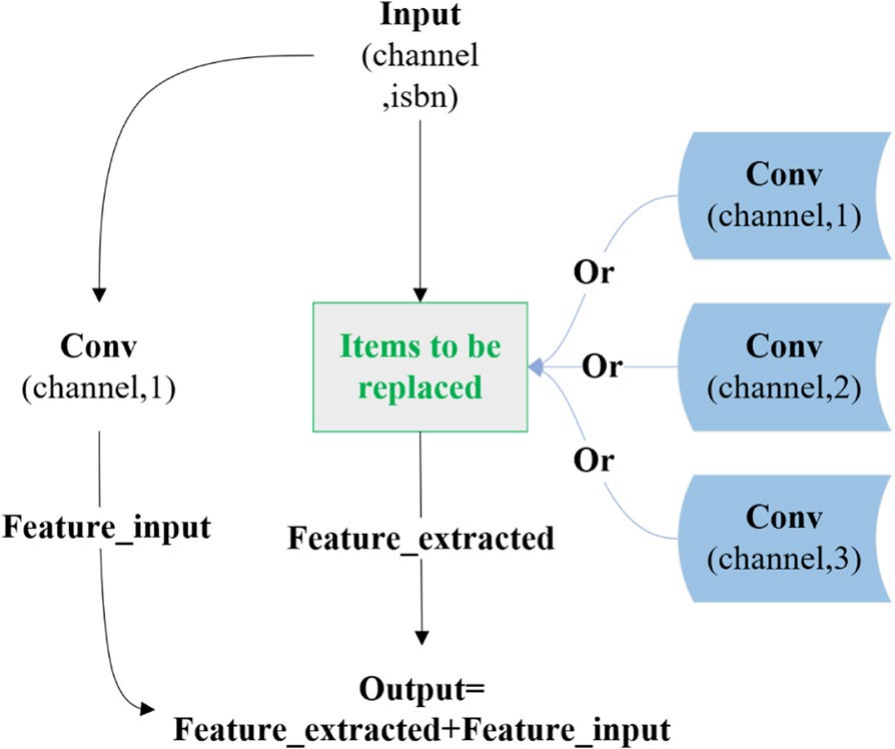
Single convolution group for replacing CCB

PointNet is a classic point cloud classification and segmentation network. The number of parameters in Gear-PCNet (${7}{\text{.89}} \times {10}^{{5}}$) is less than that of PointNet (vanilla) ($2.05 \times 10^{6}$). The effectiveness of Gear-PCNet was evaluated based on the classification performance of the three networks on gear data set. In addition, the combined CCB in Gear-PCNet was replaced with the structure shown in Fig. 9 to verify the superiority of comprehensive feature information over single feature information.

The replaced three networks were denoted: Gear-PCNet-single-1, Gear-PCNet-single-2, and Gear-PCNet-single-3. The addition of a convolution layer of kernel size 4 to the CCB in the Gear-PCNet (Gear-PCNet-4) to validate the effect of bigger kernel size on network performance was tested. The training and testing results of the above structures are presented in Table 3.

**Table 3:** Classification results of gear data set

Method	Accuracy (%)
PointNet (vanilla)	55.37
PointNet	67.31
Gear-PCNet-single-1	77.58
Gear-PCNet-single-2	78.43
Gear-PCNet-single-3	78.21
Gear-PCNet-4	80.93
Gear-PCNet	**83.42**

Table 3 shows that Gear-PCNet has the best convergence and generalization ability, and can classify and recognize each defect point of a gear with high accuracy. The results of the network with only a single convolution kernel size are inferior to Gear-PCNet verifying that the synthetic feature can more comprehensively express the information of points than a single feature. In Gear-PCNet-4, a lot of information that does not belong to the original point is extracted, and the architecture does not work well.

**Fig. 10:**
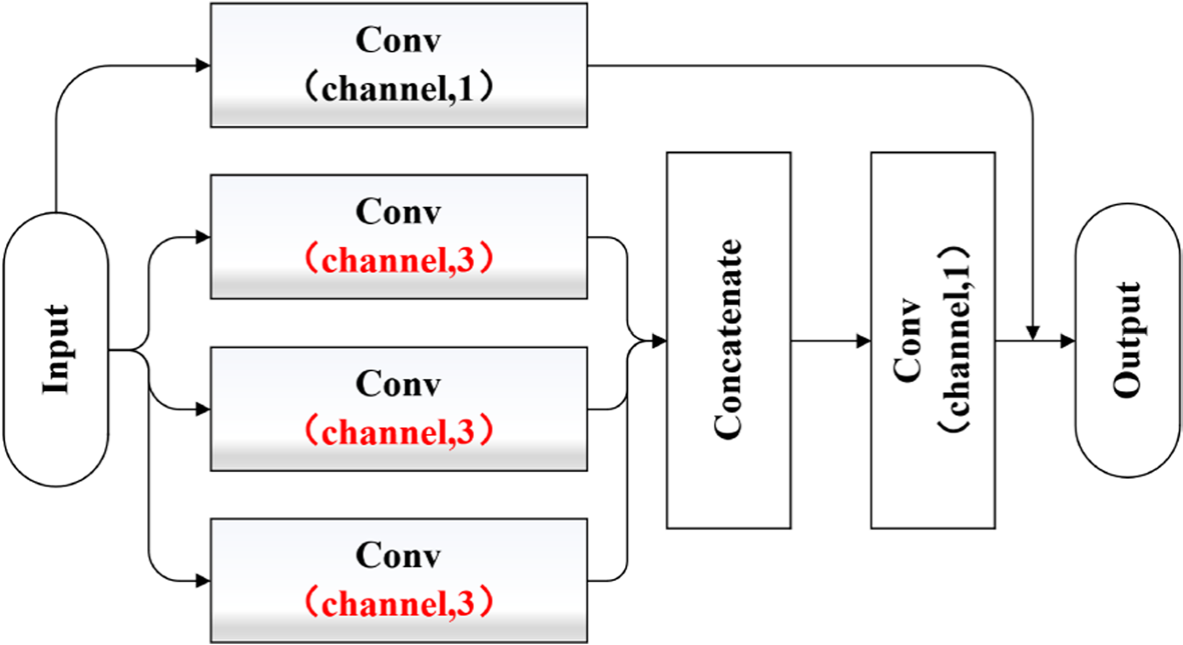
Multi convolution with single size kernel for replacing CCB

CCB was replaced with the block (Fig. 10) to verify that the better performance of the network was not due to the addition of the number of convolution layers. The testing accuracy was 78.29%, which shows the effectiveness of the CCB.

**Fig. 11:**
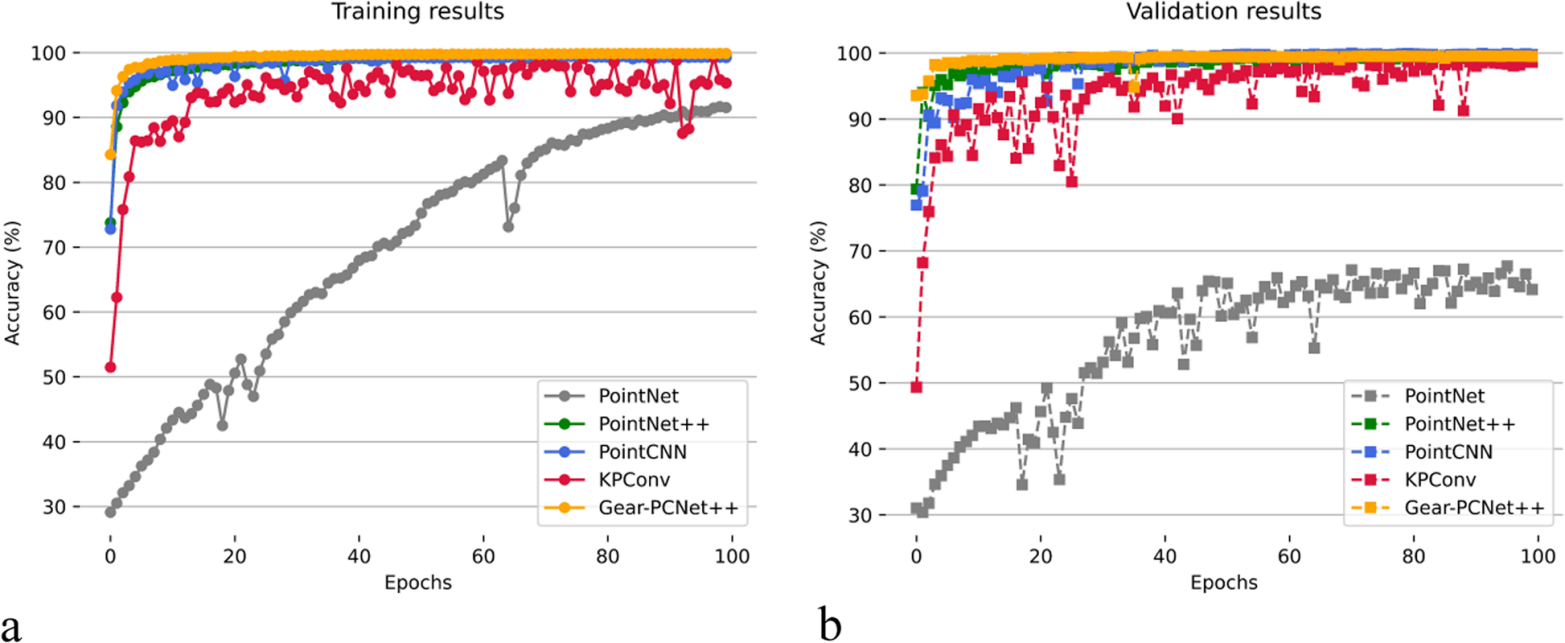
Accuracy in training and validation. **a** Accuracy of training set; **b** Accuracy of validation set. In both **a** and **b**, a marker represents the accuracy of the network at the current epoch of training or validation. Specifically, gray represents PointNet, green represents PointNet+ + , blue represents PointCNN, red represents KPconv and yellow represents Gear-PCNet++

The CCB had good results by extracting richer features. Gear-PCNet++ and several classical networks were tested on the gear data set. Figure 11 presents the prediction accuracy of the training and validation sets in the training process. It is seen that Gear-PCNet++ and PointNet++ converge faster.mAcc (mean Accuracy) and mIoU (mean Intersection-Over-Union) are the evaluation metrics; the results are listed in Table. 4. KPConv is more accurate in points classification and Gear-PCNet++ is better at object segmentation. Each architecture performs well in gear defect recognition.

**Table 4:** Segmentation results of gear data set

Method	mAcc(%)	mIoU(%)
PointNet [22]	67.31	54.66
PointNet++ [32]	99.29	98.50
PointCNN [42]	99.43	98.76
KPConv [34]	**99.64**	97.50
Gear-PCNet++	99.53	**98.97**

### Discussion of defect recognition

In Experiment results section, the classification and prediction of Gear-PCNet and Gear-PCNet++ is presented, but the types and numbers of defects in different gear models are different. In this section, the identification of defects and points in different models is analyzed based on the performance of Gear-PCNet++ on test samples (1000 gear models). Table 5 presents the recognition results of points in each model in the testing set. The recognition accuracy of 97.90% models is above 95.00%.

**Table 5:** Recognition accuracy of points in each model

Point prediction accuracy	< 85%	85%-90%	90%-95%	95%-99%	99%-100%
Proportion	0.10%	0.20%	1.80%	7.30%	90.60%
Total	2.10%			97.90%	

The judgment of defect types was considered correct if the recognition was successful, that is, if there were 3 defects in a model, if and only if the 3 defects were detected, the defect detection is considered correct. A defect existed only if there were more than 10 points labeled with the defect. Under the above settings, 99.90% models were judged correctly. This shows that the recognition results are highly reliable.

**Fig. 12:**
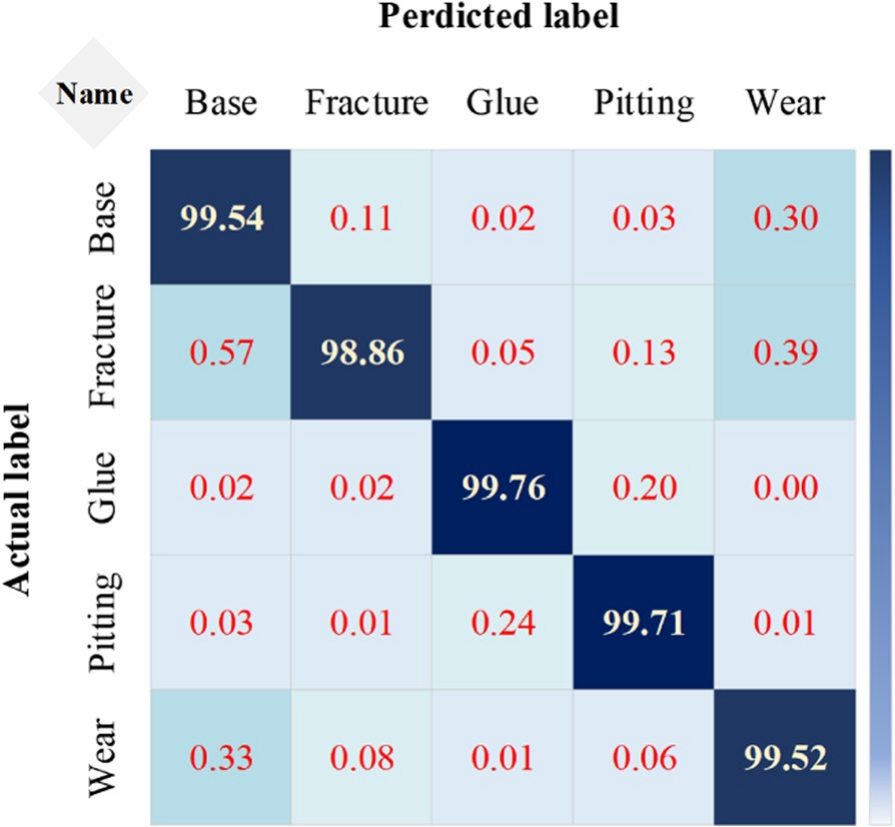
Confusion matrix of defect classification. Each row represents the distribution of predicted labels of points corresponding to each actual label. The depth of the color in the graph represents the predicted percentage

Meanwhile, Fig. 12 gives a recognition confusion matrix of each defect type in the testing set. In Fig. 12, the confusion matrix was approximated as a diagonal matrix, which also shows that the approach herein is accurate and effective.

Few recognition results of the network in this study (containing the CAD models of gears and point cloud data) given in Table 6 have the same defect color representation as Fig. 2 and defects representation as Eq. 1.

**Table 6:** Few recognition results and their original CAD models (point clouds)

	Original model	Extracted points	Predicted points	Defect recognition
Right example				Real defects: [*F*, *P*, *W*] Predicted defects: [*F*, *P*, *W*]
	Predicted accuracy of point: 100.00%			
Wrong example				Real defects: [*F*, *G*, *W*] Predicted defects:[*F*, *G*, *P*, *W*]
	Predicted accuracy of point: 96.48%			

**Fig. 13:**
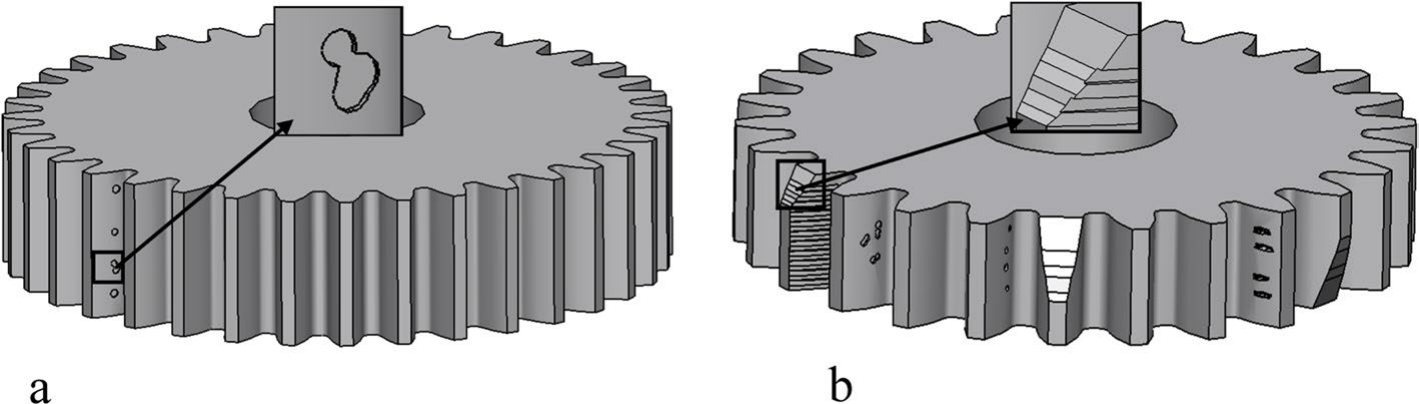
Gear models with intersecting defects. **a** Intersection result of pitting holes; **b** Intersection result of broken tooth and wear

Gears also have intersecting defects making it difficult to recognize point category. They can be divided into self-intersection of the same defect features and inter-section of different defect features. Figures. 13a and b show the intersection result of pitting holes and the intersection result of broken tooth and wear, respectively. In Gear-PCNet, these kind of intersection result may require many relevant samples to assist the training of the network; but can be satisfied in Gear-PCNet++.

## Conclusions

Gear defect recognition plays an important role in mechanical fault diagnosis. In this study, deep learning was used to extract the gear features and determine the gear defects. First, a data set of gear CAD models containing 10000 basic gears with 4 typical defects, was constructed. Second, by setting few strategies a point cloud-based gear set was generated from the gear models. Then, by giving a new CCB with three (1, 2, 3) sizes of convolution kernels, a new network: Gear-PCNet++ which can extract gear features more effectively was proposed. Finally, experimental results showed that the proposed network achieved high recognition accuracy compared to other methods for all types of gear defects.

## Data Availability

Not applicable.

## Abbreviations

CAD: Computer aided design
CCB: Combinational Convolution Block
MLP: Multi-Layer Perception
mAcc: Mean Accuracy
mIoU: Mean Intersection-Over-Union
